# Antithrombotic Management for Transcatheter Aortic Valve Implantation

**DOI:** 10.3390/jcm12247632

**Published:** 2023-12-12

**Authors:** Lina Ya’Qoub, Jelena Arnautovic, Musa Sharkawi, Mirvat AlAasnag, Hani Jneid, Islam Y. Elgendy

**Affiliations:** 1 Division of Structural Heart Disease, University of California (San Francisco), San Francisco, CA 93106, USA; 2 Division of Interventional Cardiology, Henry Ford Macomb Hospital, Clinton Twp, MI 48038, USA; jzarnautovic@gmail.com; 3 Division of Structural Heart Disease, Medical College of Georgia, Augusta, GA 30912, USA; msharkawi@augusta.edu; 4 Department of Cardiology, King Fahd Medical Center, Jeddah 21589, Saudi Arabia; mirvat@jeddacath.com; 5 Department of Cardiology, Saint Luke’s Baylor Medical Center, Houston, TX 77030, USA; hajneid@utmb.edu; 6 Division of Cardiovascular Medicine, Gill Heart Institute, University of Kentucky, Lexington, KY 40506, USA

**Keywords:** structural heart disease, transcatheter aortic valve implantation, antithrombotic, antiplatelet, anti-coagulation

## Abstract

Background: There have been significant changes in the optimal antithrombotic regimen post transcatheter aortic valve implantation (TAVI) after the results of major clinical trials in the past few years. Given the clinical importance of the optimal antithrombotic therapy post TAVI, we performed a narrative description of the major clinical trials behind the scientific evidence supporting these changes, as well the current guideline recommendations and knowledge gaps. Methods: We performed a narrative description of the major clinical trials behind the scientific evidence supporting these changes. We used PubMed as a major source to collect the major clinical trials including the following key words: “transcatheter aortic valve replacement”, “transcatheter aortic valve implantation”, “antithrombotic”, “antiplatelet” and “anticoagulation”. We selected the major clinical trials on this topic. This is not a systematic review or meta-analysis. Results: We describe the results of the major clinical trials on antithrombotic therapy post TAVI: POPULAR-TAVI A, POPULAR-TAVI B, ENVISAGE-TAVI AF, GALILEO, ATLANTIS and ADAPT-TAVR trials. Based on the results of these trials, single antiplatelet therapy is recommended post TAVI in patients without concomitant indication for oral anticoagulation or dual antiplatelet therapy, especially in elderly patients. In younger patients, it is advised to evaluate the patient’s bleeding and thrombotic risk, and dual antiplatelet therapy may be reasonable in patients with a high thrombotic risk and low bleeding risk. In patients with a concurrent indication for oral anticoagulation or dual antiplatelet therapy, it is recommended to continue oral anticoagulation or dual antiplatelet therapy post TAVI. Conclusion: In most patients without concomitant indication for oral anticoagulation, single antiplatelet therapy is recommended post TAVI.

## 1. Introduction

With the expansion in the number of TAVI procedures, understanding the optimal antithrombotic regimen for these patients is one of the key factors not only to achieve favorable clinical outcomes from the valve durability standpoint, but also to avoid peri-procedural and post-procedural complications, including valve thrombosis, stroke and bleeding [1,2,3]. Multiple clinical trials in the past decade have addressed important clinical questions and led to a change in our clinical practice [4,5,6,7,8,9,10,11,12,13,14]. In this narrative review, we discuss the major clinical trials behind the scientific evidence on antithrombotic regimens in TAVI, including antiplatelet and anticoagulation therapy, and the recommendations based on the current guidelines, as well as the knowledge gaps and future directions.

### 1.1. Background

There have been significant changes in the optimal antithrombotic regimen post TAVI after the results of major clinical trials in the past few years.

### 1.2. Methods

We performed a narrative description of the major clinical trials behind the scientific evidence supporting these changes. We used PubMed as a major source to collect the major clinical trials including the following key words: “transcatheter aortic valve replacement”, “transcatheter aortic valve implantation”, “antithrombotic”, “antiplatelet” and “anticoagulation”. We selected the major clinical trials on this topic. This is not a systematic review or a meta-analysis.

## 2. Scientific Evidence

**Figure 1:**
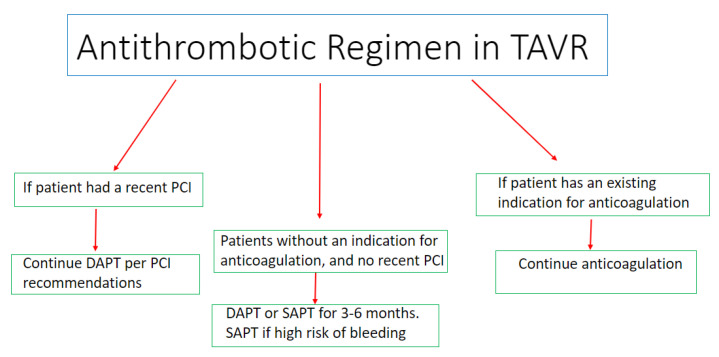
Central illustration summarizing the considerations regarding the optimal antithrombotic regimen post TAVI.

Antithrombotic therapy in TAVI is clinically indicated to prevent complications such as valve thrombosis, thromboembolic events and valve degeneration [1,2,3]. A significant proportion of TAVI patients are frail, and as such are at increased risk of both bleeding and thromboembolic events, making the risks and benefits of antithrombotic choices in this population quite challenging [1,2]. Individualized approaches of antithrombotic recommendations have been proposed based on the patients’ risk of thromboembolic events versus bleeding and the different kinds of antithrombotic medications on the market [1,2,3,4,5] [Figure 1]. Both anticoagulation therapies and antiplatelet medications have been studied in TAVI patients. In this section, we discuss the major studies evaluating antithrombotic regimens in patients undergoing TAVI.

### 2.1. Premedication in TAVI

There are multiple clinical trials assessing the different antithrombotic regimens in TAVR (Table 1) [1,2,3,4,5,6,7,8,9,10,11,12,13,14]. In the early phases of TAVI, premedication of patients undergoing TAVI was adopted; however, the safety and efficacy of the premedication approach was questioned based on the results of a few studies [1,2,3]. Loading TAVI patients with antiplatelet therapy was evaluated in a few studies [1,2,3]. The Optimized Transcatheter Valvular Intervention (OCEAN) registry was a prospective multicenter registry in Japan, which included 540 patients (80 had no pre-procedural antiplatelet therapy and 460 had antiplatelet therapy) [1]. The investigators found that patients with dual antiplatelet therapy (DAPT) had a significantly higher incidence of any bleeding than those with single antiplatelet therapy (SAPT) (36.5% vs. 27.5%, *p* = 0.049) and no antiplatelet therapy (36.5% vs. 21.3%, *p* = 0.01), without a difference in ischemic events between the groups [1]. In an analysis from the Bivalirudin instead of unfractionated Heparin in transcatheter aortic valve replacement (BRAVO 3) trial, which included 802 patients who were stratified to a clopidogrel loading dose (n = 294, 36.6%) or non-loading dose (n = 508, 63.4%) before TAVR [2], the researchers demonstrated that the loading dose of clopidogrel was associated with similar incidences of major adverse cardiovascular events (death, myocardial infarction or stroke) (4.1% vs. 4.1%, *p* = 0.97) and major bleeding (8.5% vs. 7.7%, *p* = 0.68), but a higher rate of major vascular complications (11.9% vs. 7.1%, *p* = 0.02) [2]. Based on the results of these studies, premedication with dual antiplatelet therapy or the loading antiplatelet dose before TAVI is no longer recommended and is not performed in the current clinical practice.

### 2.2. Antiplatelet Therapy in Patients without an Indication for Anticoagulation Post TAVI

Multiple studies have assessed the antithrombotic regimen post TAVI [3,4,5,6,7,8,9,10,11,12,13]. In the study by Ussia and colleagues, 79 patients were randomized to a 300 mg loading dose of clopidogrel on the day pre TAVI followed by 3 months of clopidogrel 75 mg daily plus aspirin 100 mg lifelong (N = 40) or aspirin 100 mg alone (N = 39) post TAVI [3]. The incidence of major adverse cardiac and cerebrovascular events was 14% and 16% at 30 days and 6 months, respectively, without significant differences between the DAPT and SAPT groups [3]. The Single Antiplatelet Therapy for TAVI (SAT-TAVI) was a pilot randomized study including 120 consecutive patients undergoing TAVI with a Sapien XT valve, who were randomly assigned to the DAPT group (aspirin and clopidogrel 75 mg daily or ticlopidine 500 mg twice daily) or the aspirin only group [4]. The investigators found no significant difference in the 30-day all-cause (5% vs. 5%) and cardiovascular mortality (1.7% in the DAPT group versus 3.3% in the aspirin group). However, the DAPT patients experienced higher rates of major and minor vascular complications compared with the aspirin-only group (13.3% vs. 5%; *p* < 0.05) [4]. The Aspirin Versus Aspirin + Clopidogrel Following Transcatheter Aortic Valve Implantation (ARTE) trial included 222 patients (111 patients were allocated to DAPT and 111 patients to SAPT), who underwent TAVI with balloon expandable valves [5]. The investigators found no significant difference between groups in the rates of death (DAPT, 6.3%; SAPT, 3.6%; *p* = 0.37), myocardial infarction (DAPT, 3.6%; SAPT, 0.9%; *p* = 0.18) or stroke or transient ischemic attack (DAPT, 2.7%; SAPT, 0.9%; *p* = 0.31) at 3 months [6]. They found that DAPT patients experienced a higher rate of major or life-threatening bleeding (10.8% vs. 3.6% in the SAPT group, *p* = 0.038). However, there was no significant difference in the valve hemodynamics between the two groups [5].

The Antiplatelet Therapy for Patients Undergoing Transcatheter Aortic Valve Implantation (POPular TAVI) trial cohort A, which included 665 patients undergoing TAVI without a concomitant indication for oral anticoagulation, showed that patients on aspirin monotherapy experienced significantly lower rates of all bleeding (15.1% vs. 26.6%; *p* = 0.001) and non–procedure-related bleeding (15.1% vs. 24.9%; *p* = 0.005) compared with patients who were receiving DAPT with aspirin plus clopidogrel [6]. The researchers found that this benefit was driven mainly by lower major periprocedural bleeding. There was no difference in the ischemic event rates or valve function between the two groups [6]. A meta-analysis of the four clinical trials, including a total of 1086 patients (542 patients on SAPT and 544 on DAPT) demonstrated that SAPT was associated with a lower risk of major or life-threatening bleeding without an increase in the risk of all-cause mortality, ischemic stroke or myocardial infarction [7]. Collectively, these studies showed that single antiplatelet therapy is associated with similar ischemic events post TAVI, and lower major bleeding rates, compared with dual antiplatelet therapy.

### 2.3. Antiplatelet Therapy in Patients with an Indication for Anticoagulation Post TAVI

There are two major clinical trials that assessed the addition of antiplatelet therapy to anticoagulation therapy in patients undergoing TAVR who have a concomitant indication for anticoagulation therapy; these two trials are the POPULAR-TAVI trial cohort B and the ENVISAGE-TAVI AF trial [8,9]. In the POPULAR-TAVI trial cohort B, which included 313 patients on oral anticoagulation who were randomized to clopidogrel post TAVI (157 patients were randomized to oral anticoagulation only and 156 patients were randomized to oral anticoagulation with clopidogrel) [8], the investigators demonstrated that bleeding occurred in 21.7% receiving oral anticoagulation alone and in 34.6% receiving oral anticoagulation plus clopidogrel (risk ratio, 0.63; 95% CI, 0.43 to 0.90; *p* = 0.01). Most bleeding events were at the TAVI access site [8]. Similarly, non-procedure-related bleeding occurred in 21.7% and in 34.0%, respectively (risk ratio, 0.64; 95% CI, 0.44 to 0.92; *p* = 0.02). There was no difference in the ischemic events, including cardiovascular death, stroke and myocardial infarction, between the groups [8]. The ENVISAGE-TAVI AF trial, in which 1426 patients were randomized to Edoxaban or a vitamin K antagonist, demonstrated no difference in the rates of death or stroke between the two groups, with evidence of higher gastrointestinal bleeding in the Edoxaban group [9]. In summary, these trials demostrated that patients who have an existing baseline indication for anticoagulation should continue anticoagulation alone, without the need to add antiplatelet therapy; as the addition of antiplatelet therapy is associated with higher bleeding risk, without a difference in ischemic events.

### 2.4. Anticoagulants versus Antiplatelets in Patients without an Indication for Anticoagulation

Several studies assessed antiplatelet versus anticoagulation therapy in patients undergoing TAVI who do not have a concomitant indication for anticoagulation therapy [10,11,12,13,14,15]. In the Global Study Comparing a Rivaroxaban-Based Antithrombotic Strategy to an Antiplatelet-Based Strategy after Transcatheter Aortic Valve Replacement to Optimize Clinical Outcomes (GALILEO) trial, which included 1644 patients who underwent successful TAVI, the investigators found that patients on rivaroxaban 10 mg daily in addition to aspirin for the first 3 months experienced higher rates of thromboembolic complications (hazard ratio [HR], 1.35 [95% CI, 1.01–1.81]; *p* = 0.04), death (HR, 1.69 [95% CI, 1.13–2.53]) and major, disabling or life-threatening bleeding (HR, 1.50 [95% CI, 0.95–2.37]; *p* = 0.08) compared with patients on DAPT with aspirin plus clopidogrel 75 mg daily for the first 3 months [10]. On the other hand, the analysis of the GALILEO-4D sub-study showed that patients on rivaroxaban experienced lower rates of subclinical leaflet thrombosis and leaflet motion abnormalities [11]. In the Antithrombotic Strategy After Trans-Aortic Valve Implantation for Aortic Stenosis (ATLANTIS) trial, which included 1510 TAVI patients with and without indications for oral anticoagulation, the investigators found that full-dose apixaban was not superior to standard-of-care therapy, whether it was a vitamin K antagonist or antiplatelet therapy [12]. Specifically, the investigators demonstrated that the primary outcome (composite of death, myocardial infarction, stroke or transient ischemic attack, systemic embolism, intracardiac or bioprosthesis thrombosis, deep vein thrombosis or pulmonary embolism and life-threatening, disabling or major bleeding) occurred in 18.4% and 20.1% patients receiving apixaban or the standard of care, respectively (HR 0.92; 95% CI 0.73–1.16). Major or life-threatening bleeding was similar between the groups (HR 1.02; 95% CI 0.72–1.44). However, valve thrombosis was lower in the apixaban group versus antiplatelet therapy (HR 0.19; 95% CI 0.08–0.46) [12].

Similarly, in the FRANCE-TAVI registry, which included a total of 12,804 patients in the registry, the researchers demonstrated that despite the fact that patients on full-dose oral anticoagulation on discharge, mainly with vitamin K antagonists, had lower rates of bioprosthetic valve dysfunction and subclinical leaflet thrombosis, oral anticoagulation therapy was associated with a significantly higher risk of death, independently of atrial fibrillation and other comorbid confounders (adjusted HR, 1.25 [95% CI, 1.08–1.44]; *p* = 0.002) [13]. In the ADAPT-TAVR trial, patients receiving edoxaban therapy experienced a lower incidence of leaflet thrombosis compared with patients on dual antiplatelet therapy (9.8% vs. 18.4%; 95% CI, −17.8% to 0.8%; *p* = 0.076) [13]. However, there was no significant difference in new cerebral thromboembolic events and neurological or neurocognitive function between the two groups [14]. A meta-analysis of the three major trials (ATLANTIS, GALILEO and ADAPT-TAVR trials) assessing direct oral anticoagulation versus antiplatelet therapy in TAVI patients without a pre-existing indication for anticoagulation therapy demonstrated a higher all-cause mortality and non-cardiac mortality in the direct oral anticoagulation group compared with the antiplatelet group [15]. The investigators found no difference in the bleeding and thromboembolic events between the two groups [15]. Collectively, current evidence from these trials does not support the use of anticoagulation after TAVI unless there is a concomitant indication for anticoagulation.

### 2.5. Pharmacodynamics and Antiplatelet Therapy in TAVI

Little is known about the pharmacodynamic effects of different antithrombotic strategies in patients undergoing TAVI [16]. In the REAC-TAVI (Assessment of Platelet Reactivity After Transcatheter Aortic Valve Implantation) trial, which included 68 patients on DAPT with aspirin and clopidogrel and 48 patients with high platelet reactivity who were randomized to ticagrelor or clopidogrel for 3 months [16], all patients receiving ticagrelor had reduced platelet reactivity compared with only 21% of patients who received clopidogrel having reduced platelet reactivity, suggesting ticagrelor is more efficacious at reducing platelet reactivity compared with clopidogrel [16]. Importantly, the prevalence of high platelet reactivity was 71% in this study cohort, suggesting that high platelet reactivity is probably common in TAVI patients. Although high platelet reactivity is a well-known risk factor for stent thrombosis in patients undergoing percutaneous coronary interventions, its clinical and prognostic implications in patients undergoing TAVI remain uncertain [16]. In addition, the pharmacodynamic effect of the lower ticagrelor dose (60 mg twice daily) is currently unknown and is being studied in the Platelet Reactivity According to Ticagrelor Dose After Transcatheter Aortic Valve Implantation (REACTIC-TAVI) trial (NCT04331145), which includes 40 patients with high platelet reactivity.

## 3. Current Guidelines

There are differences in the recommendations between the American and European guidelines [Table 2]. The American College of Cardiology/American Heart Association (ACC/AHA) published the most recently updated guidelines on antithrombotic therapy for patients undergoing TAVI in 2021 [17]. Based on the 2021 ACC/AHA guidelines, for patients undergoing TAVI and who are at low risk of bleeding, DAPT with aspirin 75–100 mg daily and clopidogrel 75 mg daily may be reasonable for 3–6 months after valve implantation in patients, with a class of recommendation (COR) IIB, level of evidence (LOE) B-NR [17]. The guidelines provided a COR IIA, LOE B-NR for aspirin 75–100 mg daily as a reasonable option in patients undergoing TAVI in the absence of other indications for oral anticoagulation [17]. The guidelines recommend the use of novel oral anticoagulation as an effective alternative to vitamin K antagonists for patients with an elevated CHA_2_DS_2_-VASc score and atrial fibrillation who received a bioprosthetic valve (COR I, LOE A) [17]. For patients with TAVI at a low risk of bleeding, the use of vitamin K antagonists to achieve an INR of 2.5 may be reasonable for at least 3 months (COR IIB, LOE B-NR) [17]. For patients with TAVI, treatment with low-dose rivaroxaban (10 mg DAILY) plus ASA (75–100 mg/d) is contraindicated in the absence of other indications for OAC (COR III, LOE B-R) [17].

The 2021 European Society of Cardiology/European Association of Cardiothoracic Surgery (ESC/EACTS) guidelines recommend lifelong single antiplatelet therapy (aspirin 75–100 mg daily or clopidogrel 75 mg daily) after TAVI in patients with no baseline indication for oral anticoagulation (COR I, LOE A) [18,19]. Routine use of oral anticoagulation is not recommended in patients with no baseline indication for OAC (COR III, LOE B) [18]. The ESC guidelines recommend oral anticoagulation lifelong for TAVI in patients who have other indications for oral anticoagulation (COR I, LOE B) [18].

## 4. Current Knowledge Gaps and Future Directions

There are limited data about the clinical implication of subclinical leaflet thrombosis (SLT) and how to prevent and manage this condition [20,21]. SLT is usually diagnosed using contrast-enhanced computed tomography demonstrating hypo-attenuated leaflet thickening, reduced leaflet motion and mild elevated valvular gradients on echocardiography [20,21]. In recent studies, SLT occurred in up to a quarter of patients on antiplatelet therapy [20,21]. Oral anticoagulation prevents and treats leaflet thrombosis [20]. Although observational studies showed an association between SLT and thromboembolic events, these findings were not confirmed in clinical trials, including the GALILEO and ADAPT-TAVR trials [10,14,20,21]. It is important to note that the investigators in the ADAPT-TAVR trial found a trend towards lower leaflet thrombosis in the edoxaban group; however, there was no difference in neurological events assessed by magnetic resonance imaging of the brain, as well as serial neurological and neurocognitive functional testing between the edoxaban and the DAPT group [14]. The clinical impact of SLT on valve function and durability remains unclear at the current time.

For patients undergoing valve-in-valve TAVI, the risk of clinical valve thrombosis is higher than native valve TAVI (7% vs. 1%), making this population at an especially increased risk for thromboembolic events [22]. Factors that potentially affect the thrombotic risk include the surgical or transcatheter valve type, post-deployment hemodynamics and valve geometry, patient–prosthetic mismatch and flow stasis [22]. Although oral anticoagulation lowers the risk of valve thrombosis, the benefit of routine anticoagulation in these patients is unknown and is not recommended by the current guidelines [17,18,22].

Furthermore, a single antiplatelet strategy using potent P2Y12 inhibitors alone post TAVI has not been studied, which represents an important knowledge gap. The mechanism of action of different P2Y12 inhibitors, including clopidogrel, ticagrelor and prasugrel, in TAVI patients is not well-understood, and the effect of potent P2Y12 inhibitors could be potentially more beneficial than aspirin. Additionally, data on the specific outcomes of different antithrombotic therapies in different kinds of prosthetic valves are lacking. Given the scarce data on the efficacy and safety of these different medications in different prosthetic valves post TAVI, studies investigating the outcomes of potent P2Y12 inhibitors in different kinds of prosthetic valves post TAVI are encouraged. In fact, the pharmacodynamic effect of the lower 60 mg dose of ticagrelor monotherapy post TAVI is currently being investigated in the REACTIC-TAVI trial (NCT04331145).

Similarly, there are limited data on the use of novel anticoagulation therapy post-surgical aortic valve replacement, while there are multiple trials showing the safety of using novel anticoagulation medications post TAVI [10,11,12,13,14,16,17,22]. Although analysis of the available data showed non-inferior outcomes of using novel anticoagulation therapy post-surgical aortic valve replacement, only a small proportion of the available data compromises the first 3 months post-surgical aortic valve replacement, which is a major knowledge gap and future studies are highly encouraged in this field [23].

In this context, it is important to individualize care for patients undergoing TAVI; as we assess the thrombotic versus bleeding risk. Scoring systems might be useful in that regard, similar to the thromboembolic (CHADVASC score) and bleeding (HAS-BLED) risk tools used in patients with atrial fibrillation [24]. Although we do not have a formal bleeding and thrombotic risk tool dedicated for TAVI patients at the present time, many TAVI patients share similar risk factors for bleeding and thromboembolic events. As such, clinicians might extrapolate the existing tools such as CHADVSAC and HAS-BLED scores in a non-atrial fibrillation population [24]. Furthermore, incorporating thrombocytopenia post TAVI in these clinical scores is important as thrombocytopenia post TAVI is not uncommon and is potentially associated with sepsis and acute kidney injury post TAVI. Studies have shown that thrombocytopenia post TAVR may be related to worse short- and long-term outcomes [25]. In addition, bleeding and thrombotic risks post TAVI should be assessed longitudinally, i.e., patients may become at a higher risk of bleeding months after TAVI, or they may develop new-onset atrial fibrillation post TAVI, thus emphasizing the importance of assessing these changes in clinical status longitudinally in order to provide the optimal recommendation regarding the antithrombotic regimen at different time points for our patients post TAVI.

## 5. Conclusions

The optimal antithrombotic regimen for TAVI has evolved in the past decade based on the results of several randomized clinical trials. Based on our current clinical practice, single antiplatelet therapy is recommended post TAVI in patients without a concomitant indication for oral anticoagulation or dual antiplatelet therapy, especially in elderly patients who are 80 years old or older. This is mainly to decrease bleeding complications in these elderly patients. In patients who are 79 years old or younger, it is recommended to evaluate the patient’s bleeding and thrombotic risk, and dual antiplatelet therapy may be reasonable in patients with a high thrombotic risk and low bleeding risk. In patients with a concurrent indication for oral anticoagulation or dual antiplatelet therapy, it is recommended to continue oral anticoagulation or dual antiplatelet therapy post TAVI. Additionally, multiple ongoing trials are underway to understand the current knowledge gaps, including subclinical leaflet thrombosis, bleeding and thrombotic risk tools and the clinical outcomes of different P2Y12 inhibitors in different kinds of prosthetic valves in patients undergoing TAVI.

## Figures and Tables

**Table 1:** Summary of the major randomized clinical trials assessing the antithrombotic regimen following TAVI.

Study, Year	Number of Patients	Antithrombotic Regimen	Major Findings
POPULAR-TAVI, cohort A, 2020 [6]	665	Aspirin 80–100 mg daily versus 3 months of DAPT with aspirin 80–100 mg daily + clopidogrel 75 mg daily	Aspirin monotherapy was associated with lower bleeding (15.1% vs. 26.6%; *p* = 0.001) and non–procedure-related bleeding (15.1% vs. 24.9%; *p* = 0.005) than DAPT; There was no difference in ischemic event rates and valve function between the groups
POPULAR-TAVI, cohort B, 2020 [7]	313	Oral anticoagulation alone versus oral anticoagulation with clopidogrel 75 mg daily for 3 months	Bleeding occurred in 21.7% receiving oral anticoagulation alone and in 34.6% receiving oral anticoagulation plus clopidogrel (*p* = 0.01); There was no difference in the ischemic events, including cardiovascular death, stroke and myocardial infarction, between the groups
ENVISAGE-TAVI AF trial, 2021 [8]	1426	Edoxaban versus vitamin K antagonist	No difference in the rates of death or stroke between the two groups, with evidence of higher gastrointestinal bleeding in the Edoxaban group
GALILEO, 2020 [9]	1644	Rivaroxaban 10 mg daily (with aspirin 75–100 mg daily for 3 months) versus aspirin 75–100 mg daily (with clopidogrel 75 mg daily for 3 months)	Rivaroxaban 10 mg/d (plus aspirin for the first 3 months) was associated with a higher risk of thromboembolic complications and death compared with DAPT
ATLANTIS trial, 2022 [10]	1510	Full dose apixaban versus vitamin K antagonist or antiplatelet therapy	Full dose apixaban was not superior to standard-of-care therapy, whether it was a vitamin K antagonist or antiplatelet therapy
ADAPT-TAVR, 2022 [13]	229	Edoxaban versus DAPT	There was a trend toward a lower incidence of leaflet thrombosis in the Edoxaban group compared with the dual antiplatelet therapy (9.8% vs. 18.4%; *p* = 0.076). No difference in new cerebral thromboembolism and neurological or neurocognitive function between the groups

**Table 2:** Summary of the current guidelines on antithrombotic therapy recommendation post TAVI.

Guidelines	Recommendation	Class of Recommendation	Level of Evidence
2021 ACC/AHA	DAPT with aspirin 75–100 mg daily and clopidogrel 75 mg daily may be reasonable for 3–6 months after valve implantation in patients at a low risk of bleeding [17]	IIB	B-NR
	SAPT with aspirin 75–100 mg daily as a reasonable option in patients undergoing TAVI in the absence of other indications for oral anticoagulation [17]	IIA	B-NR
	Novel oral anticoagulation as an effective alternative to vitamin K antagonist for patients with an elevated CHA_2_DS_2_-VASc score and atrial fibrillation who received a bioprosthetic valve [17]	I	A
	Vitamin K antagonists to achieve an INR of 2.5 may be reasonable for at least 3 months in patients with low risk of bleeding [17]	IIB	B-NR
	Low-dose rivaroxaban (10 mg daily) plus ASA (75–100 mg daily) is contraindicated in the absence of other indications for oral anticoagulation	III	B-NR
2021 ESC/EACTS	Lifelong SAPT (aspirin 75–100 mg daily or clopidogrel 75 mg daily) after TAVI in patients with no baseline indication for oral anticoagulation	I	A
	Oral anticoagulation lifelong for TAVI in patients who have other indications for oral anticoagulation	I	B
	Routine use of oral anticoagulation is not recommended in patients with no baseline indication for OAC	III	B
